# Antigenic Protein Screening and Design of Multi‐Epitope Vaccine Against *Lactococcus garvieri* and *Streptococcus iniae* for Combating *Lactococcosis* and *Streptococcosis* in Fish

**DOI:** 10.1002/vms3.70465

**Published:** 2025-06-17

**Authors:** Ramesh Ranjbar, Abbas Doosti, Mostafa Shakhsi‐Niaei

**Affiliations:** ^1^ Department of Biology ShK.C. Islamic Azad University Shahrekord Iran; ^2^ Biotechnology Research Center ShK.C. Islamic Azad University Shahrekord Iran; ^3^ Department of Genetics Faculty of Basic Sciences Shahrekord University Shahrekord Iran

**Keywords:** Lactococcus garvieae, Streptococcus iniae, immunoinformatic, multi‐epitope vaccine

## Abstract

The illness caused by *Lactococcus garvieae* and *Streptococcus iniae* is well acknowledged as a disease that results in significant economic losses since it affects a diverse array of fish species. The constraints of existing vaccinations and techniques have prompted the exploration of novel approaches to manage this ailment. Multi‐epitope vaccines that use a diverse range of immunogenic proteins have considerable potential.

The primary objective of the present research endeavour was to develop a very effective multi‐epitope vaccine targeting *Streptococcus iniae* and *Lactococcus garvieae* infection in fish.

The immunogenic components of *Lactococcus garvieae* and *Streptococcus iniae* were used for epitope prediction. A multi‐epitope vaccine was constructed using the immunogenic proteins' most effective B cell epitopes and the GFFY adjuvant. Subsequently, an assessment was conducted on many aspects of the developed vaccine, including physicochemical characteristics, antigenicity, secondary structure and tertiary structure. Furthermore, the molecular docking technique was used to study the interaction between the proposed vaccine and its TLR‐5 receptor. The nucleotide sequence of the vaccine was subsequently modified to facilitate its expression in *Lactococcus lactis*.

The findings of the current investigation indicate that the vaccine developed exhibited stability, as shown by its molecular weight of 93989.19 Da and antigenicity value of 0.8547. In addition, the study of the vaccine's structure indicated that it consisted of 32.24% alpha helix, with 88.41% of its residues located in the preferred area. The proposed vaccine effectively docked to its TLR5 receptor was shown, resulting in the lowest energy of ‐995.4.

According to the data obtained, the developed vaccine has the potential to effectively prevent infection in fish caused by *Lactococcus garvieae* and *Streptococcus iniae*. Our findings suggest that the peptide vaccine might be a favourable choice for prophylaxis against *Lactococcus garvieae* and *Streptococcus iniae*.

## Graphical abstract

The image depicts the computational identification of B cell epitopes from *Lactococcus garvieae* and *Streptococcus iniae*, leading to the design of an epitope‐based vaccine for fish. It features a stylised fish with the targeted bacteria and illustrates the process of identifying B cell epitopes using bioinformatics tools. The vaccine design is also shown, ready for administration to the fish.

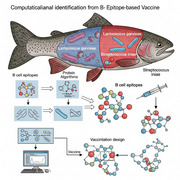

## 1 Introduction

Over the last two decades, there have been intentional deliberations about the concept of sustainability in aquaculture on a global scale (Irshath et al. 2023). Aquaculture is now recognised as the most rapidly expanding food industry worldwide (Irshath et al. 2023; de Bruijn et al. 2018), boasting an output of 85.3 million tonnes in 2019. It substantially contributes to nutrition and food security, especially in places with the highest levels of food insecurity, while sustaining millions of people's livelihoods globally. Aquaculture accounted for 46% of worldwide fish output in 2018, with Asia being the dominant region in quantity and economic value, accounting for 80% of global aquaculture production. According to the United Nations Food and Agricultural Organisation (FAO), it is projected that a 70% augmentation in the global food and feed supply would be necessary to sustain the growing human population by the year 2050. Due to the growth of the human population and the rising affluence of individuals in the Asia‐Pacific area, there is an anticipated 30% increase in the demand for aquaculture by 2030 (de Bruijn et al. 2018). Infections in fish that lead to disease outbreaks are a severe problem for the aquaculture industry due to the potential for substantial economic harm caused by illness and mortality. High fish‐rearing density in aquaculture facilitates the transmission and dissemination of harmful microbes, often as a critical catalyst for catastrophic outbreaks (Rohani et al. 2022).

Numerous bacterial illnesses have been documented in fish species within the aquaculture industry, such as *Lactococcosis* (Khalil et al. 2023), *Edwardsiellosis* (Armwood et al. 2022), *Streptococcosis* (Van Doan et al. 2022) and *vibriosis* (Xu et al. 2022). However, it has been shown that a small number of these pathogens significantly contribute to the decrease in aquaculture output worldwide (Verdegem et al. 2023). *Lactococcosis* and *Streptococcosis* are the most important bacterial diseases caused by *Lactococcus garvieae* and *Streptococcus iniae* bacteria (Brunt and Austin 2005).

Numerous fish species are infected by the facultative anaerobic, non‐motile, Gram‐positive *Lactococcus garvieae* bacterium, which also causes significant financial losses in freshwater and marine aquacultures. *Lactococcus garvieae* has been associated with the occurrence of deadly haemorrhagic septicaemia and has been shown to induce meningoencephalitis in many fish species and other animal species (Vendrell et al. 2007). This bacterium is a nascent fish disease that impacts a diverse array of fish in freshwater and marine environments, resulting in substantial financial repercussions within the aquaculture sector throughout several regions, including the Mediterranean, Japan, Europe, Southeast Asian nations and North America. The first isolation of *L. garvieae* occurred in clinical samples of bovine mastitis within the United Kingdom area, followed by its simultaneous isolation from yellowtail (*Seriola quinqueradiata*) in Japan. Over the last several decades, warm water lactococcosis, caused by *L. garvieae*, has emerged as a significant and lethal disease in the farmed production of rainbow trout throughout the summer months when water temperatures is above 21°C (Irshath et al. 2023; Halimi et al. 2020).

Fish infections produced by streptococcal bacteria have resulted in high death rates in wild and farmed fish, leading to substantial financial losses for the aquaculture sector. *Streptococcus iniae* is a prevalent bacterial pathogen often linked to outbreaks of *streptococcosis* in farmed fish (Liu et al. 2019). *Streptococcus iniae* has a wide distribution among its hosts and has been seen to impact a minimum of 27 fish species, including a significant proportion of commercially significant species, including rainbow trout, salmon, channel catfish and Japanese flounder (Irshath et al. 2023; Sun et al. 2013).

Fish infections persist as a significant economic concern in the global commercial aquaculture sector despite several endeavours to advance novel therapeutic approaches (Irshath et al. 2023; Rahimi et al. 2010). While antibiotics may be utilised in treating fish diseases, they are accompanied by evident drawbacks, including drug resistance and issues about consumer safety and environmental impact (Manyi‐Loh et al. 2018). Vaccination is a very effective strategy for mitigating a wide range of viral and bacterial illnesses, significantly contributing to the aquaculture industry's overall sustainability worldwide (Ma et al. 2019). Despite the existence of several bacterial vaccines that are commercially accessible for aquaculture production, the development and manufacture of effective vaccinations for various bacterial illnesses remain a challenge (Ben Hamed et al. 2021). The progress in molecular biology, biotechnology and reverse vaccinology has enabled the development of various types of vaccinations, such as subunit vaccines, plasmid DNA immunisations, recombinant live vector vaccinations and recombinant vaccines. These vaccines have undergone experimental testing in fish and have received approval for commercial use (Taherzadeh‐Soureshjani and Chehelgerdi 2020). A subunit vaccine was developed for fish nocardiosis in the largemouth bass (Micropterus salmoides) using a reverse vaccinology examination of specific pathogens' effective antigenic target contents. The results showed that the vaccines showed great promise for nocardial prophylaxis despite varying effects (Ho et al. 2018).

There is a lack of comprehensive vaccinology research that can assess and compare antigenic antigens' potential and advantageous qualities as possible vaccine candidates. Hence, the present research used a distinctive reverse vaccinology procedure to choose the most suitable sequence among the constructed entities. This approach allowed the comparison of various synthesised molecules via computer methodologies. The generated structures were subjected to docking and molecular dynamics (MD) modelling and comprehensive analysis of their physicochemical characteristics, secondary and tertiary constructions, and conformational epitopes. The present investigation facilitated the identification of promising vaccine candidates targeting *Streptococcus iniae* and *L. garvieae*, contributing to time and cost savings.

## 2 Materials and Methods

### 2.1 Genomes Retrieval, Process of Protein Screening and the Prediction of Allergenicity

The genomes of *Lactococcus garvieae* and *Streptococcus iniae* strains were acquired from the GenBank database using the National Centre for Biotechnology Information (NCBI) for computational study (https://www.ncbi.nlm.nih.gov/). The genomes, both in their whole and partly obtained, were converted into the FASTA version. If the BLASTp website validates the sensitivity of the proteins, *Streptococcus iniae* and *Lactococcus garvieae* proteins will be utilised for further studies. The UNIPORT dataset was used to investigate the proteomics of *Lactococcus garvieae* and *Streptococcus iniae* to discover extracellular proteins. The NCBI has uploaded the amino acid compositions of these proteins in FASTA format for future research. The VaxiJen v2.0 webserver was employed to evaluate the protective antigens, and a threshold value of 0.45 was selected for each.

### 2.2 Epitopes Prediction

#### 2.2.1 Linear B Cell Lymphocytes (LBL)

B cell epitopes are necessary to enhance humoral or antibody‐mediated defences. To anticipate the linear B cell lymphocyte (LBL) epitopes, we employed the IEDB server (http://tools.iedb.org/main/bcell/) with default settings. The expected epitopes were evaluated using the VaxiJen v2.0 and AllerTop v2.0 platforms.

#### 2.2.2 The Construction of a Multi‐Epitope Vaccine

The vaccine was developed by combining the selected LBL epitopes with an appropriate adjuvant and connecting them with the appropriate linkers. The adjuvant utilised in this study was a TLR5 agonist since extramembrane proteins can identify TLR5. Adjuvants play a crucial role in overcoming the limitations of translation and synthesis. Consequently, the immunogenicity of the vaccine candidate was assessed by evaluating the GFFY tetrapeptide adjuvant, an artificial Toll‐like receptor‐5 agonist. This phenomenon facilitates stimulating the immune system's innate and adaptive components. Toll‐like receptors and antigen‐presenting cells (APCs) initiate innate immunity. Employing the EAAAK linker facilitated the connection between several bacterial antigens and adjuvants. Conversely, the chosen antigens were connected using AAY linkers, while each antigen's epitopes were connected using the KK linker. The AAY linker is a location of proteasome cleavage used to alter the stability of proteins, reduce their immunogenicity and enhance the presentation of epitopes. Using a bi‐lysine KK linker in the vaccine design preserves the distinct immunogenic characteristics.

#### 2.2.3 Analysis of the Vaccine's Structure

The essential features of a protein are described by its physiochemistry. The ProtParam webserver (https://web.expasy.org/protparam/) was used to anticipate the physicochemical characteristics of the vaccination in order to acquire a thorough grasp of its crucial function. The immunological qualities were assessed using advanced platforms such as VaxiJen v2.0 (http://www.ddg‐pharmfac.net/vaxijen/VaxiJen/VaxiJen.html) and SOLpro (https://protein‐sol.manchester.ac.uk/). The self‐optimised prediction method with alignment (SOPMA) website (https://npsa‐prabi.ibcp.fr/cgi‐bin/npsa_automat.pl?page = /NPSA/npsa_sopma.html), along with the PSIPRED v4.0 website (http://bioinf.cs.ucl.ac.uk/psipred/), utilises default settings to identify the two‐dimensional (2D) structural elements of the construct, including the κ‐helix, β‐turn, and random coils. The prediction accuracy of SOPMA exceeds 80%. In order to get a deeper understanding of the vaccine's compositional quality, we obtained and evaluated 2D structural parameters.

#### 2.2.4 Forecasting and Verification of Tertiary Structure

The developed vaccination was sent to the RaptorX platform via the URL, http://raptorx6.uchicago.edu/ContactMap/. The RaptorX website utilises an advanced algorithm and a three‐dimensional structure to generate the most accurate protein structure and its corresponding activities. This online tool can predict and calculate the C‐score, TM‐score significance, RMSD, and top five models of a specific protein sequence. The produced three‐dimensional structure was stored as a protein data bank (PDB) file, selected depending on the C‐score. The server's C‐score varies between ‐5 and 2, with a higher value suggesting greater confidence in the protein model. The vaccine structure was refined by uploading the identified 3D structure to the GalaxyRefne online web‐based platform (http://galaxy.seoklab.org/refne). The web server was operated using the CASP10 refining technique. The GalaxyRefne website provides access to the RMSD, energy score, and overall quality score. The enhanced framework was acquired and the selected framework was identified based on the energy scores of the minimum and maximum root mean square deviation (RMSD) values. The revised and found structure was shown using PyMOL v2.3.4. In the final 3D structure analysis, the Ramachandran plot score was used to assess the validity of the vaccine structure. Additionally, the Z‐score value was utilised to quantify the standard deviation from the mean value. The Ramachandran plot was analysed using the SWISS‐MODEL service (https://swissmodel.expasy.org/), which examines the areas of permitted and banned amino acids. The Z‐score plot was analysed using ProSAweb (https://prosa.services.came.sbg.ac.at/prosa.php/). The stereochemical quality of a protein structure was assessed using UCLA‐DOE LAB — SAVES v6.0 (https://saves.mbi.ucla.edu/), which included the analysis of residue‐by‐residue geometry and overall structure geometry.

#### 2.2.5 Discontinuous B Cell Epitope Prediction

In almost 90% of instances, it was shown that B cell epitopes exhibited discontinuity. The 3D architectures of discontinuous (conformational) B‐cell epitopes have been modelled using the computer tool ElliPro (http://tools.iedb.org/ellipro/). ElliPro employs three techniques to ascertain the protein structure as an ellipsoid, the residue PI, and the nearby cluster residues based on the protrusion index (PI) values. The ElliPro software computes the mean PI quantity for every output epitope by summing the residues of each epitope. 90% of the amino acid residues in the ellipse with a PI value of 0.9 are inside the spheroid, whereas the remaining 10% are outside. The principal intensity (PI) score for each antigenic residue was calculated by considering its location relative to a maximal ellipsoid of residue mass. According to our findings, ElliPro is the most effective structure‐based strategy for epitope prediction, with an AUC value of 0.837, surpassing all other protein prediction methods we evaluated.

#### 2.2.6 Disulfide Engineering of the Vaccine Construct

In order to progress and start docking analysis, the developed model must exhibit stability. Disulfide bonds contribute to the structural stability of proteins. The bonds for the suggested vaccine were assigned using Disulfide by Design 2.0 (http://cptweb.cpt.wayne.edu/DbD2/index.php).

#### 2.2.7 Molecular Docking Analysis

Molecular docking studies may be used to elucidate the chemical reactions that occur between modelling proteins and receptor molecules. To submit the revised vaccination model as a binding compound and the TLR5 molecule as an immunological receptor for molecular docking, the ClusPro v2.0 website (https://cluspro.org/help.php) was utilised. The TLR5 ligand was selected and obtained from the Protein Data Bank (PDB) website with the PDB ID: 3V44. The first stage in receptor preparation was ligand extraction from the peptides, followed by the elimination of water and other chemical substances. The operations above were executed with the PyMOL v2.3.4 software. The binding connections and residues in the contacting region were investigated using Discovery Studio 2017 (https://discover.3ds.com/discovery‐studio‐visualizer‐download) and PDBSum (https://www.ebi.ac.uk/thornton‐srv/databases/pdbsum/). The molecular docking and refinement processes were conducted using the online server ClusPro v2.0. The docking process was repeated for the third iteration utilising the HawkDock server (http://cadd.zju.edu.cn/hawkdock/). Subsequently, the molecular mechanics/generalised born surface area (MM‐GBSA) score was computed using the identical server that predicts the affinity score outcome. The score with the lowest prediction was considered superior.

#### 2.2.8 MD Simulation Analysis

The iMODS modelling website (https://imods.iqfr.csic.es/), which is open‐source in nature, provides a user‐friendly interface for doing internal normal mode analysis (NMA) on the given protein. This study aims to assess the protein's deformability, stability and motility. Individuals can do NMA or simulate possible pathways connecting two conformations, therefore seeing the resulting results in three dimensions, even when dealing with many biomolecular complexes. The iMODS method was used to evaluate the dynamic stability of the TLR5‐vaccine complexes in this investigation.

#### 2.2.9 Codon Adaptation and In Silico Cloning

Codon optimisation is essential for expressing a foreign gene in a host. Consequently, the construct was submitted to the JCat service platform for codon modification (http://jcat.de/). The *Lactococcus lactis* strain was used as the host organism in this investigation. The whole technique was conducted with careful consideration of three specific criteria:

- The avoidance of places where restriction enzyme cleavage occurs.
- The avoidance of binding sites on prokaryotic ribosomes.
- The avoidance of rho‐independent transcription termination.

The modified sequence's evaluation included using the codon adaptation index (CAI) score and the guanine‐cytosine (GC) concentration. Finally, the changed nucleotide sequence was used to clone the adapted nucleotide pattern into the pNZ8124 expression plasmid by in silico methods. The complete in silico cloning technique was conducted using SnapGene v4.2 software. The RNAfold website (http://rna.tbi.univie.ac.at/cgi‐bin/RNAWebSuite/RNAfold.cgi) was also used to evaluate expressed mRNA sequences' thermodynamic dependability and translation efficiency.

## 3 Results

### 3.1 Analysis of the Proteins

The subcellular localisation of the proteins and the associated transmembrane helices is anticipated. The subcellular location is crucial in developing medicines, vaccines and transmembrane helices. We made predictions on the subcellular distribution of proteins in both the inner and outer membranes. Moreover, the expected transmembrane helices are predicted are predicted. The Vaxijen website is used to predict antigenicity. Furthermore, the proteins under investigation exhibited poor results in terms of allergenicity and toxicity. Table 1 displays the proteins that were examined at this stage.

**TABLE 1:** Initial identification and analysis of proteins.

					Protein‐sol	
Pathogen	Protein	ACCESSION	Sequence	VaxiJen	Solubility	pI
*Lactococcus garvieae*	cell surface protein	UHU65762.1	>UHU65762.1 cell surface protein [*Lactococcus garvieae*] MKKINIGFVLLLLLLNTASPAVYALDSETEIQSSSSATSDKDNTSSPQESELETAPTDTGQTTEAENSTD QTSEEVAPIEEEEPTTSETSEQEKTVEDKPKSEKITPADLSSDASYTDLPGAHKPDRNGLYLLFPLRTGI LSQPQEEQYIVEGTAADLSIKTTSWVNTWGTVKVKVNAWQEQADGSWLNKGSIGEFSDRSGILGSTKQSI DIPVSKLQAGNYYFQLETRLNQVPIIGLAATYYSDLAKVTVKDKPVAASGISITTPNVVFEKATYSAKAV TTPRDATGKITWTSSLKGINFLKTEGRSVEFDAGDQLDEVNTDEQAPGIPFTMSAGIRNDDGTTPTDNKT IYLGGLAAKNVAEDSGMSWELDGQGLADLNKSVESAETWSYEWRYIEGKTSKAFSETQGVKNYKGTVSDL TSLNDADHILSFAQDNAFMKTAREATEKGKPFAVQVTFSTTIDTGDGKKETVTLSSNKAQLLVNKATGKL LLQQVPNFNFGKLPASYIYEGTKDREPIAQDHLEVVDSRAKGGWRLSAKMSRFQSNNNQRLKFNSQLTMS SYPFEINLKDDDTDNYIVDSVSSEAFDLSGQLLLQPDPLVDLNAGETFSSTITWNLVSTTKSTPPA	0.7009	0.719	4.570
	Anionic cell wall polymer biosynthesis enzyme, LytR‐Cps2A‐Psr (LCP) family	SFL21325.1	>SFL21325.1 Anionic cell wall polymer biosynthesis enzyme, LytR‐Cps2A‐Psr (LCP) family [*Lactococcus garvieae*] MKDNKQKRFEYLRANKAYLSEREKQEYYELVEEIEGWQALEEEYAADLEEERAQSESSSPERRSRSSRSL DAAPAAFLSQRGKKKESRRAEENTNDETSEETASKKPKKKKRWIKRIFFLLIALILIMIGFFIYGYQRGI SREGGAIKAEQFNGAANADGSVNILLLGADQRPGQSSGVAHSDSIMVLNIGKSGKMQLVSFMRDTLVNIP GVGDASQGPNMKLNAAFTIGEQNENQGVELVRQTLQQNFGINAKYYAVVDFSSFATVIDSMFPGGVQIDA KFATINGQKFDEVPVPDDLAETEGMAKNDKELSAEEAAALGYPDGGGIFMMIKQGPQRMDGRTLLNYARF RHDDQGDFGRVQRQQQVMQTLTSKVKNPLTLFTGASAMGTARAVTMTNLPNTFLLTKALPAMFGGIENTT IPSDNDWQSAYDMYGGSGILIDMNKYSAKAQELLGQ	0.6351	0.463	5.510
	WxL domain surface cell wall‐binding	SFL13552.1	>SFL13552.1 WxL domain surface cell wall‐binding [*Lactococcus garvieae*] MKTKKIISLTALALIASSTGASTVFAADGGVYNSDSTITYTPASGPTDPVDPGDPGTTVDPEGPKNPGTD GPLSIDYASNFNFGTQEITSADKTYFAAATTLTDKTTRPNWVQVTDNRGTLAGWSLSLQASEFTNGKTGT GSVLSGATLKLENGHIVSASDAAADQSVASVTLTPGTSSGTILGATAGKGAGTNLLVWGDDTTKATSVSL AVPGKTTKLADTYQSTLTWTLTDTPAN	0.7377	0.672	4.490
	T surface‐antigen of pili	OAL08711.1	>OAL08711.1 T surface‐antigen of pili [*Lactococcus garvieae*] MKTLRQVLKGLVLAIVLSVLIISGMRLKNTLSRVSADRVDKKTDQKVAPIRDNNNNTAQILEKEAWWTDQ EAYEANLLLKVNGSSLSGPMDVVFVLDRSGSMDMTYTDNANATGYEGYPLFSSSCLNQEHFYLEPLHEGQ EPEASADKSKVYENSDNTLTVYNADVDKWEVIGTTPVHLFEQFTQSTAKYIPYHFKKEGEKFVRISHWDA AAKVAGKTQPGVWVHGDEDEGCYDRWMLSKEAISEVTDKLLQEHPENRVALAPFSIRDSSMVTHLNYNSQ RMKNFRDNLVLKNNEHGYFGPSTGTTIEADGSIGGNYNSTVGWKDNIAENRAAIDDMLPRLFTTPQTDYQ YGLSMAYNLLQSRSDEAKATKGAMVVFLSDGVPQSTATMRAGWSGGGPIVSFGQTDGRILAMSAAITSDE PVEVEGAPTGTYQRHDIDQNYLKLIEGTTYEVAGMGAEMLTVDYMANSAILKTMANDPQNYFEVPADNVG AGKTYLTDLLLNSTLFPGGRDSVLRDKVSQYYYVPENATLPQGVTIEGNYEDKGEEQTIVWELGDLYDYS PDGYPSVNIPLVLKEEYRQVSQDTYYPTNADTSDKAMDILDPERGIDDEDTGAKLYYTDPHNQKRYDTIG TPKLAVKPDTVKSTRYQIQAEKLLSGRSLLAGEFAFELLDSAGKVIQTVHNDASGKIEFSPITYTSTGDY DYTVREKGGLDKTIDYDAVNHKVTVSIKEEAGNLVATATGDAAIQFKNSYQPLGTEIHLQATKELVGKKL IDQEFTFELVNASNQVIQTAKNDALGQIRFDKLAYTKTGSYDYTIREKAGVDPQITYDTKSYKIHVEVVD QKGKLVASISYKQKPQFKNIYLEEKKSSSKDDGNKNLIEKVEPQKKVLPKAGEFNKTAAIVTGLGLILMV LAIFWARKFRKNRK	0.5929	0.507	5.180
	competence type IV pilus major pilin ComGC	WP_311799472.1	>WP_311799472.1 competence type IV pilus major pilin ComGC [*Lactococcus garvieae*] MEKKRFKAFTLIEMLVVLLIISVLLLLFVPNLSKEKKSIQNTGQTAVVKVVEGQAELYQLDKQDSPSLGK LVSGGLITQKQADSYNDYYTKNPNEKRNVPN	0.5401	0.753	9.800
	FctA domain‐containing protein	WP_197909309.1	>WP_197909309.1 FctA domain‐containing protein [*Lactococcus garvieae*] MKHLRKVLKVFTLVMITIGLFATGLLYKNSTKQHVSADKVKQAAVQKEDPIRDKGSNTARIVEKEAWWTN QEAYEANLLLKVNGSSLSGPMDVVFVLDRSGSMDMTYTDNANIAGYEGYPLFSSSCLNQDHFYLEPLRQT SEPEANADKAKVYTNNDNTLTVYNADVDQWEVIGTTPVHLFEQFTQSTAKYIPYHFKMEGGNYVRISHWD AGAKVSGKAEPGVWVHGDESEGCYDRWMLSKEAITEVTSNLLQEHPENRVALVPFSIRDSTMLEHLNYNS ERMKNFRDNLVSQNAAKGYFGPSTGTSVDASGTIVGSYNSTVGWTNSVAENKAAMEDMLPRLFTTPQTDY QYGLSMAYNLLQSRSDTAKATKGAMVVFLSDGVPQSTATMRVGWAGGGGIVSFGQTDGRILAMSEAITSN DPVQIEGEPSGTYQRHDIDTSYLSHVNGSTYEAAGMGAEMLTIDYMANSAILKSMANDPQNYLEVPADNV GAGKTYLSDLLLNSTLFPGGRESVLRDKVSKYYYVPENATLPQGVTIEGNYEEKGEEQTIVWDLGDLYDY APDGYPTINIPLVLKDEYRQVAQDTYYPTNADTSEKGTDIYDSERGIDDEDTGAKLFYKDPHNQNRYDTI GTPKLAVVPDPSKSASYQIQAAKHLSGRSLLADEFTFELLDDTGQVIQTATNDAAGQVVFDKIEYTSVGD YAYTIREKTGLDKTIDYDSASHQVTVHVKEENGQLLASATGESEAAFRNSYQPISTTIDFKATKELTGKK LQDKEFTFELLDDTDKVIQTATNDTSGQIHFDKISYTKAGNYDYVIREQQGKDAQITYDSSAYKIHVKVE DKKGQLVATPTYEHPLHFKNIYHTPEKTTPPQSGDTFSLVKIKNIWKEVTKTTDQMLPETGEGSTKAVAT LGIILIVLALFTFGVTKFRGRKR	0.5951	0.530	5.000
*Streptococcus iniae*	cell wall surface anchor family protein	ASL34282.1	>ASL34282.1 cell wall surface anchor family protein [*Streptococcus iniae*] MEREIMKKFNKVKVAESFQKTRVKMHKSGKRWIRTIMSHVGLIHLFKGGRDESDIQSDNLERGGISSATV LKGVAALGTLAAGGSLGVDKISAEDSVLVSDASSSTFTGTDVVLMASGSYTNGRAATLNTSVPKSSTAAS YVSKAGVQLESLLIVDSGFTPVLKGIQFISTTTNFVTVAEAVSYLQAHPNAIGTMKYYYEIIDNSTGTTH VLNDGQAEVGNLNYQITNIAPVVSKIDIYTWRGTQKYVEYPASDPDGTISNVIFKNANNTNTSNNIGNLA FSVQNGKVIYSGSTTSTTKLGRYPESIDVVDNAGAITNSGTIYIYVLDASGGSITKEWNQTVTEQEILDK ATILAGDSTVPTTIQKKLISPIPTYNPQSKTTTVQVQLTTPDGEVKVVDVIVTYNDATSISASESLSISE SFSIAESVSESTSESILESVSESTSESVIESVSESVSESHSESLSESISESTSDSLSESISESTSESLSE SISESVSESLSESVSESTSESLSESISESTSESLSESISESTSESLSESISESTSESLSESISESTSESL SESISESTSESLSESISESTSESLSESISESTSESVSESISESTSESLSESISESTSESLSESISESTSE SLSESISESTSESLSESISESTSESLSESISESTSESLSESMSESTSESLSESISESTSESLSESISESV SESLSESISESTSESLSESISESVSESLSESVSESTSESVSESISESTSESLSESISESTSESLSESISE SVSESLSESVSESTSESLSESISESTSESLSESISESTSESLSESISESTSESLSESISESTSESLSESI SESTSESVSESISESTSESLSESISESMSESLSESISESISESLSESISESTSESLSESISESTFESLSE SISESTSESLSESISESVSESLSESVSESTSESVSESISESTSESLSESISESTSESLSESISESTSESL SESISESTSESLSESISESTSESLSESISDSTSESLSESISESTSESLSESISESTSESLSESISESTSE SVSESISESTSESLSESISESTSESLSESISESTSESLSESISESTSESVSESISESTSESLSESISESV SESLSESISESTSESLSESISESTSESLSESISESVSESLSESISESTSESVSESISESTSESLSESISE STSESLSESISESTSESLSESISESTSESVSESISESTSESLSESISESTSESLSESISESTSESLSESI SDSTSESVSESISESTSESLSESISESTSESVSESISESTSESLSESISESTSESLSESISESTSESLSE STSESVSESISESTSESLSESISESTSESVSESISESTSESLSESTSESVSESISESTSESLSESISEST SESLSESISESTSESLSESISESVSESLSESISESTSESLSESISESTSESGWAKPLPHTGEESASIATA LGAGLVFAGLFGRKKKKEKDFEHSLSESISESTSELLSESITDSLIEAFSELASESMTESSQSNELS	0.7173	0.777	3.610
	SEC10/PgrA surface exclusion domain‐containing protein [*Streptococcus iniae*]	WP_121792243.1	>WP_121792243.1 SEC10/PgrA surface exclusion domain‐containing protein [*Streptococcus iniae*] MELENKSKNIKTTIALTSTLALLSASVGVAQQVKAEEASAQKNGMNTSSDETMAMPTTVEDARTAVATTE ATLSAQNTNLSKVNAEIDATNKELLTLEAKKAEEAAALAAAQKTLETVSAVSEKEFTALVEKNKEELTTT KAQLQQNEAEKLAVASRVQKQTELVATTGAEAKKLAEQAAQADKKVSDLTKMVNQPEKITAQAHEAEKDL ATTTSELAKAKANLTAVTEAAKKQLNQELATNQATLANKQAELTKLQHTATSTRINVVGNNKMVIPNGFP FAEIQKLMASGYIGSQSYLNAFNSMKDTLIARTSPGQAINTYVDIPSDLNRFVNIDQLTPEVQNELALFA TSMINSVRQQLGLSQLIVSQGSQDFARYLTTSYKATHGNTRPFFNYGQAGVAGHRGIGPHDKTIIQASAQ KVGLIPNDDNMYENLGFFDDVHTVNGIKRSIYNSVRYMLFTDYLHGNTLGHAVNFLRWDKTNPSAPVYLG VSTSSVGGLNTHYVVFPKTNIKNPSAFNQSLVSGPTITVSNASQINNLKTSIANLNTKISSLKQRIAHVS SEAQVVSAQNRVNSYKVQNDFAKTELAKLKGQLVQLKQSKTKLESDLASAKTLQRSVKAQLDQKLAFLTT AKAQLNTFNNQLHLSNVKVVGLVSKQKELQALLDFKNNPNRKEMAKVDVAKMQTELAATTAKLDISKNNL ASLMSRKTRLLSAITSTEQQLRLLQNVVKEKTVLIPGPLGLTAHPEAVAEVVASKILEAKAASPVAALKT TEIKKEQVSESKTDKTANLVAQTTTELVKDALAVSPQILAGQGILAKVADNISKGTDSTNLGYGSGSTLA GDMALSNDESTKRAIRAGVVMLTAVGLTGFKLKKDIK	0.4905	0.545	9.740
	MetQ/NlpA family ABC transporter substrate‐binding protein	WP_016356113.	>WP_016356113.1 MetQ/NlpA family ABC transporter substrate‐binding protein [*Streptococcus iniae*] MKVNKLFSLLGLVAVSTLLVACSGKQDDKNTLTVGVMTKTDSDQARWDKIEELLKKENITLKYKEFTDYS QPNKAVANGEVDINAFQHYNFLNNWNKENKENLVAIADTSISPIHLFSGTDQKGKAKYKTVEDLPKGAQI AVPNDATNESRALYLLEAVGLIKLNVSGEKLATIANVTENKKDLDIKELDASQTARSLTSADAAIVNNSF AVPAKIDYKTSLYKEAVDKNTNQWINVIAGKKDWKKSDKADAINKLVKVYHTDDVKKVVDKTSNGIDIAV W	0.4942	0.784	9.000
	metal ABC transporter substrate‐binding protein	RLV17335.1	>RLV17335.1 metal ABC transporter substrate‐binding protein [*Streptococcus iniae*] MFKKISLAFAMLLSIFCITACSSQQQASKDKKLDVVVTNSIIADMTKNIAGKKINLHSIVPIGQDPHEYE PLPEDVEKTTNADLIFYNGINLEDGGQAWFTKLVKNAKKTKNKDYFAVSDGIDVIYLEGENEKGKEDPHA WLNLENGVIYSKNIAKQLMAKDPENKDYYQKNLDAYVAKLEKLDQEAKSAFDKIPDNKKVIVTSEGCFKY FSKAYKVPSAYIWEINTEEEGTPDQISSLIEKLKAKKPSALFVESSVDSRPMKSVSKDSGIPIYSEIFTD SVAKKGQDGDSYYAMMKWNLDKISEGLAK	0.5307	0.617	5.950
	CHAP domain‐containing protein	RMI67436.1	>RMI67436.1 CHAP domain‐containing protein [*Streptococcus iniae*] MKKDKYIALLMLSALLLPSFNAVSVVANETSTATNQEAVTSITDLEKDSAKESAVSEAKEVTPVVEQEVP LPQEPAKDGTSTDATKPGEVTEPSKPVEPSIPKPDQGQKDPEKPGDTTSPVQPPVVSVPTTPVVPATPAV SEAPTTAPIQAPVLPITAPISKFTAMESRPSASAFAAYVDHWTDSDAYTHNLLSRRYGIKAEQLDGFLQS TGIAYDSKRINGQKLLDWEKESGLDVRAIVAIALAESSLGTQGVAKTPGANMFGYAAFDHSPQSAQQFSD DIAIIKLTQETIVQNHNSSFAIQDKKAQLLSDGLLNTSVDGGVYFTDASGTGKRRAEVMEKLDQWIDRHG GTPEIPAELRVQASASLASVPVGYKLSKLNRVIDYISSTYAWGQCTWYVYNRGQELGYQFDAYMGNGGDW KMKPGYGVTHKAEVGYAVSFSPGQAGADPTYGHVAIVEEVKKDGSILISESNALGLGVVSYRTFTAAEAA QLTYVVGHK	0.4918	0.555	5.020
	cell surface protein	OHX27211.1	>OHX27211.1 cell surface protein [*Streptococcus iniae*] MKIKKQNEKGHGYFRKSKAYGLVCGIALAGVFALSGGNVLAEEVTAPTSEVVTTLTTPTNNQAQPVTSSE LDKAVEEAKDAGVNVTTTAPVAHVDVSTAQTDLANQNKAVEEATAKAEANATAIKTATDANAKIDAENDA EAKRVAEANKAGQLATDQKNAEAKAKVDQANKDAQAQADATNAKLKAEYEAKLTEIKTVEDYNKAVAERN ALAKQQADATNAQLKADYQTKLDAYNKALADKVNLIANDVSFEGYGKSEVLSAYGTVTSDTVSIDNAGNF TLREPQTDQYGIFGYITTKGKLNYSSVYDTATNKAKITIDSITLGTWQLDLDRPSRAGNTNVSAEYIALD GTSLFKQAFTNLTSIAPITIGKTSAIGQSFVLASGETTPEFMFLKTNPYWEFFAPSSLYAKLTYKTASLP ENPTPPVYKTVTPEAEKPVPQIPPVPVYVTPELKSFTPEVYTPIKPTVKPHVSVPEKLTVTVSVHPVLVP VANPSKDVVDEAGKSINGLSVLPNTDLNYVAKQDFSQYKGMTANKDAVAKNFIFIDDYKDEALDGKSMTV NSIKASNGQDVSNLLEMHHVLSKDALDAKLQAIIAQSGLSPVGEFYIWVAKDPASFYQAYVQKGLDITYN LSFKVNQTFAEGQIVNGVAQIDFGNGYLGNLVINDLPKPEVHKDVLDKQDGKSINNGTVKLGDEVTYKLE GWVIPTGRSYDLFEYKFVDQLQHSHDLYLKDSVVAKVDITLTDGTVIAKGSNLAQYTETIYNKETGCYEL VFKKDFLAKVARESEFGADDFITVKRVKAGDVYNDYTLYVNGNPVLSNKVVTHTPEKPQPPKPTTPEKML PHAGEQSMGLLTAVGAGIISILGLAGLKRKEGK	0.5874	0.557	5.490

### 3.2 LBL Epitopes Prediction

**FIGURE 1:**
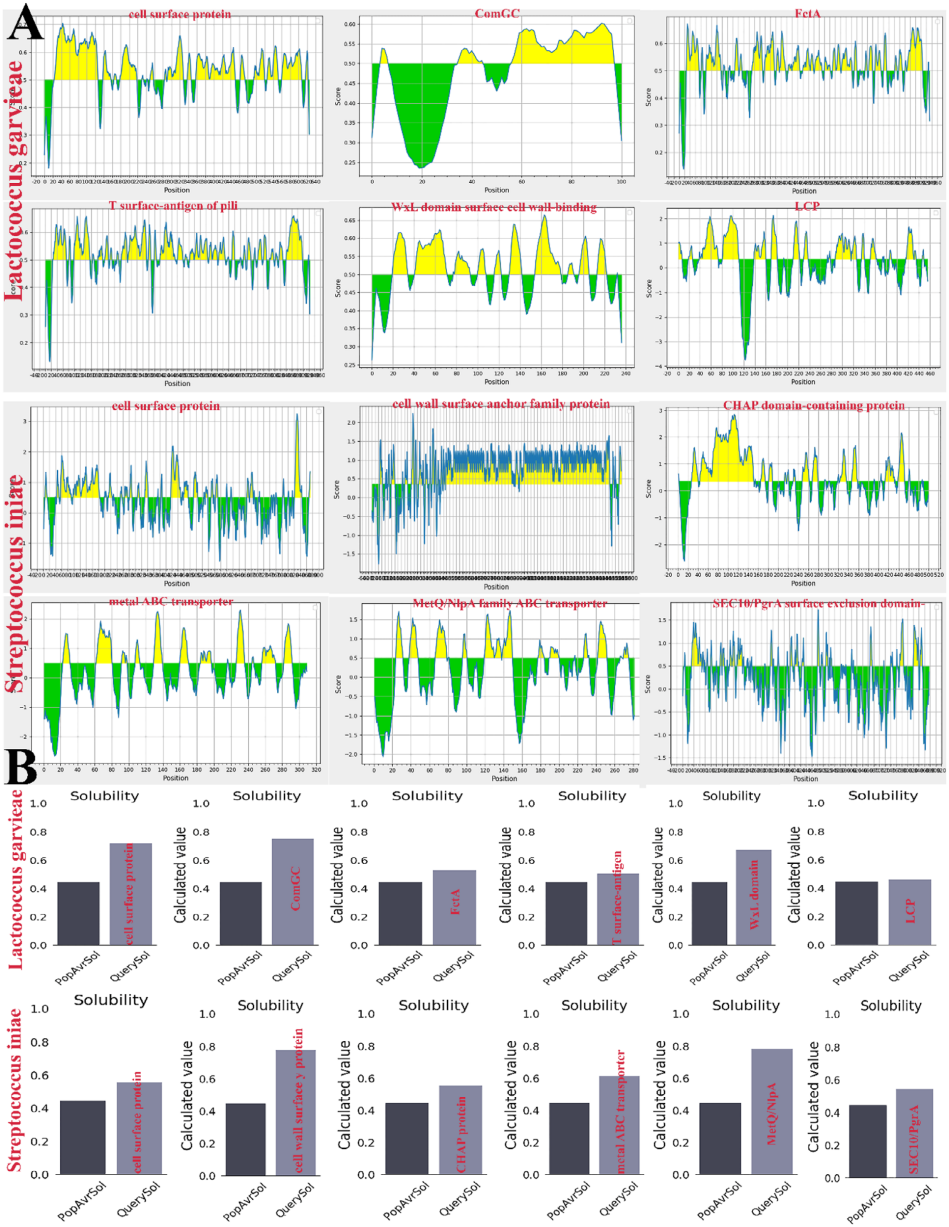
(A) Prevalence of *Lactococcus garvieae* and *Streptococcus iniae* protein LBL epitopes. (B) Solubility of *Lactococcus garvieae* and *Streptococcus iniae* selected proteins.

Figure 1A presents the prediction of distinct LBL epitopes, considering their toxicity, immunogenicity, antigenicity and non‐allergenicity. For the final assembly of the vaccine, the epitopes that exhibited the highest likelihood, antigenicity, allergenicity and toxicity were selected. Figure 1B shows the solubility of the corresponding proteins. The epitopes located in the outer part of the cell were selected as the final epitopes. The study conducted by IEBD was used to select B‐cell linear epitopes with a score of 0.5 for vaccine design. Table 2 presents data about the quantity of surface‐exposed B‐cell epitopes. It includes the epitope sequence and adjusted rank of each linear epitope.

**TABLE 2:** Evaluation of LBL epitopes according to their positioning in the cellular outer layer.

Pathogen	Protein	Start	End	Epitope	Epitope outer part	
					Start‐end	Sequence
*Lactococcus garvieae*	cell surface protein	25	129	LDSETEIQSSSSATSDKDNTSSPQESELETAPTDTGQTTEAENSTDQTSEEVAPIEEEEPTTSETSEQEKTVEDKPKSEKITPADLSSDASYTDLPGAHKPDR	25‐129	LDSETEIQSSSSATSDKDNTSSPQESELETAPTDTGQTTEAENSTDQTSEEVAPIEEEEPTTSETSEQEKTVEDKPKSEKITPADLSSDASYTDLPGAHKPDR
		250	258	TVKDKPVAA	250‐258	TVKDKPVAA
		266	279	GIRNDDGTTPTDNK	266‐272	GIRNDDG
		311	319	KSVESAETW	311‐319	KSVESAETW
		391	404	TIDTGDGKKETVTL	396‐404	DGKKETVTL
		633	639	VSTTKST	633‐639	VSTTKST
	Anionic cell wall polymer biosynthesis enzyme, LytR‐Cps2A‐Psr (LCP) family	17	117	AYLSEREKQEYYELVEEIEGWQALEEEYAADLEEERAQSESSSPERRSRSSRSLDAAPAAFLSQRGKKKESRRAEENTNDETSEETASKKPKKKKRWIK	17‐117	AYLSEREKQEYYELVEEIEGWQALEEEYAADLEEERAQSESSSPERRSRSSRSLDAAPAAFLSQRGKKKESRRAEENTNDETSEETASKKPKKKKRWIK
	WxL domain surface cell wall‐binding	21	33	ASTVFAADGGVYNS	21‐33	ASTVFAADGGVYNS
		29	75	GGVYNSDSTITYTPASGPTDPVDPGDPGTTVDPEGPKNPGTDGPLSI	42‐71	PASGPTDPVDPGDPGTTVDPEGPKNPGTDG
		78	93	ASNFNFGTQEITSADK	78‐93	ASNFNFGTQEITSADK
		101	123	TLTDKTTRPNWVQVTDNRGTLAG	101‐108	TLTDKTTR
					119‐125	NRGTLAG
		159	183	HIVSASDAAADQSVASVTLTPGTSS	159‐183	HIVSASDAAADQSVASVTLTPGTSS
		185	192	ATAGKGAG	187‐191	AGKGA
	T surface‐antigen of pili	102	159	GSMDMTYTDNANATGYEGYPLFSSSCLNQEHFYLEPLHEGQEPEASADKSKVYENSDNTL	102‐157	GSMDMTYTDNANATGYEGYPLFSSSCLNQEHFYLEPLHEGQEPEASADKSKVYENSD
		600	643	LDPERGIDDEDTGAKLYYTDPHNQKRYDTIGTPKLAVKPDTVKS	606‐644	IDDEDTGAKLYYTDPHNQKRYDT
					636‐641	VKPDTV
		817	830	EKAGVDPQITYDTK	817‐829	EKAGVDPQITYDT
	competence type IV pilus major pilin ComGC	57	109	LYQLDKQDSPSLGKLVSGGLITQKQADSYNDYYTKNPNEKRN	57‐109	LYQLDKQDSPSLGKLVSGGLITQKQADSYNDYYTKNPNEKRN
	FctA domain‐containing protein	103	160	GSMDMTYTDNANIAGYEGYPLFSSSCLNQDHFYLEPLRQTSEPEANADKAKVYTNN	103‐160	GSMDMTYTDNANIAGYEGYPLFSSSCLNQDHFYLEPLRQTSEPEANADKAKVYTNN
		473	516	LTIDYMANSAILKSMANDPQNYLEVPADNVGAGKTYLSDLLL	473‐516	LTIDYMANSAILKSMANDPQNYLEVPADNVGAGKTYLSDLLL
*Streptococcus iniae*	cell wall surface anchor family protein	19	31	QKTRVKMHKSGKR	19‐31	QKTRVKMHKSGKR
		910	1466	ESISESTSESLSESISESVSESLSESVSESTSESVSESISESTSESLSESISESTSESLSESISESTSESLSESISESTS………LSESISESTSESLSESISESTSESGWAKPLPHTGEESAS	Repetitive sequence	SESISESTSESLSESISES
	SEC10/PgrA surface exclusion domain‐containing protein	17	22	TSTLAL	17‐20	TSTL
		118	130	ETVSAVSEKEFTA	118‐126	ETVSAVSEK
	MetQ/NlpA family ABC transporter substrate‐binding protein	38	48	TKTDSDQARWD	39‐47	KTDSDQARW
		96	101	NKENKE	96‐100	NKENK
		119	149	GTDQKGKAKYKTVEDLPKGAQIAVPNDATNE	120‐129	TDQKGKAKYK
					132‐138	VEDLPKG
					139‐149	QIAVPNDATNE
		177	184	VTENKKDL	177‐184	VTENKKDL
		189	200	LDASQTARSLTS	190‐199	ASQTARSLT
		264	275	DVKKVVDKTSNG	270‐275	DKTSNG
	metal ABC transporter substrate‐binding protein	61	81	PIGQDPHEYEPLPEDVEKTTN	62‐80	IGQDPHEYEPLPEDVEKTT
		107	113	AKKTKNK	108‐113	KKTKNK
		183	197	LDQEAKSAFDKIPDN	184‐196	DQEAKSAFDKIPD
	CHAP domain‐containing protein	439	452	SFSPGQAGADPTYG	444‐451	GQAGADPTY
		27	40	ANETSTATNQEAVT	27‐40	ANETSTATNQEAVT
	cell surface protein	4	14	KKQNEKGHGYF	6‐12	QNEKGHG
		290	303	REPQTDQYGIFGY	294‐303	QTDQYGIFGY
		41	51	AEEVTAPTSEV	43‐51	EVTAPTSEV

### 3.3 The Composition of a Vaccination and Its Core Properties

**FIGURE 2:**
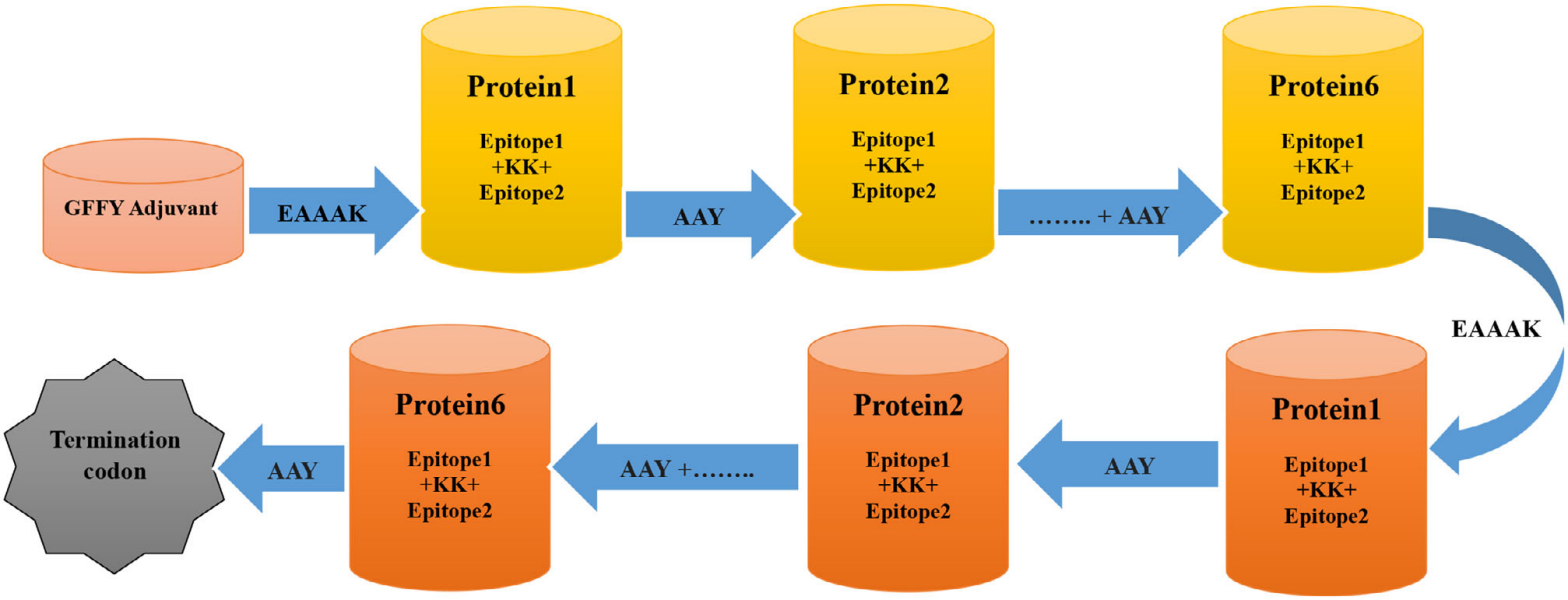
Schematic sequence of the constructed vaccine.

The vaccine was constructed using previous selections of LBL epitopes. The linkers KK and AAY were employed to connect epitopes. An adjuvant was used in front of the construct to increase immunogenicity. An adjuvant was linked to the LBL epitope using the GFFY sequence to activate TLR5. The final vaccine had 288 amino acids in total (Figure 2).

### 3.4 Evaluation of the Immune System and Analysis of the Physical and Chemical Properties

The physicochemical properties of the vaccine construct are shown in Table 3. The molecular weight of the vaccine construct was determined to be 93989.19 Da. Additional characteristics were observed, including a theoretical isoelectric point (pI) of 6.32, the chemical composition C4059H6454N1126O1415S10, an instability index of 44.22, an aliphatic index of 48.13, and a grand average hydropathicity of ‐1.166. For mammalian reticulocytes grown in vitro, the predicted half‐life was 30 h. The extinction coefficients are given in units of M^−1^ cm^−1^, observed at a wavelength of 280 nm in water. The vaccine construct had an extinction coefficient of 80695. Additionally, the construct's physicochemical attributes and immunological efficiency were evaluated. For example, the construct's antigenicity was determined to be 0.8541, while its immunogenicity was found to be positive. Furthermore, the vaccine demonstrated solubility, scoring 0.643 out of 1.

**TABLE 3:** The construct exhibits distinct characteristics regarding antigenicity, allergy and physicochemical qualities.

Row	Characteristics	Finding	Remark
1	Number of amino acids	856	Suitable
2	Molecular weight	93989.19 Da	Average
3	Theoretical pI	6.32	Acidic
4	Chemical formula	C_4059_H_6454_N_1126_O_1415_S_10_	—
5	Instability index of vaccine	44.22	Stable
6	Aliphatic index of vaccine	48.13	Thermostable
7	GRAVY	−1.166	Hydrophilic
8	Antigenicity	0.8547	Antigenic
9	Immunogenicity	Positive	Immunogenic
10	Allergenicity	No	Non‐allergen
11	Solubility	0.643	Soluble

**FIGURE 3:**
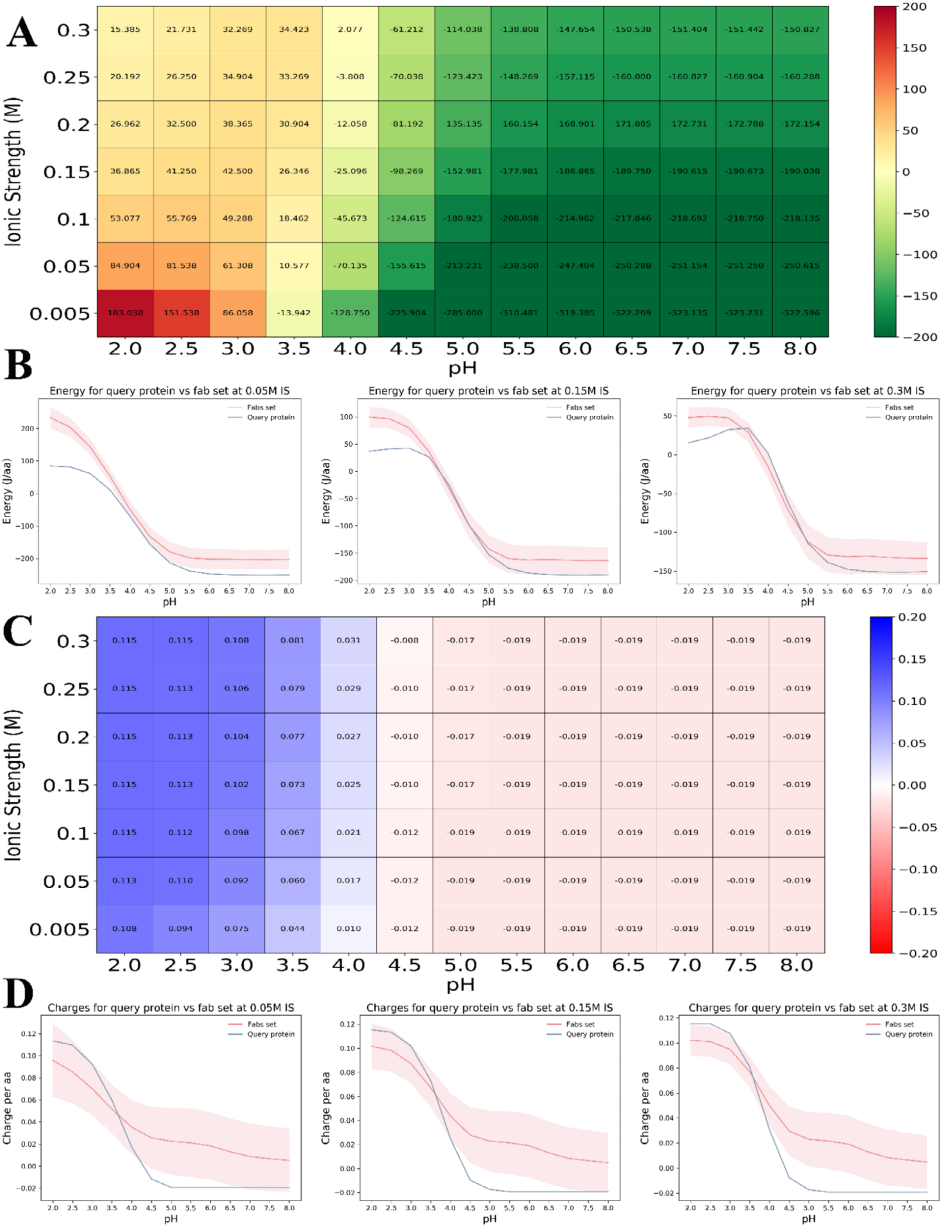
Web‐based display of protein surface and pH‐dependent properties for assessing the developability of biotherapeutics. (A) The vaccine structure's energy heat map illustrates the accurate distribution of energy. (B) Energy of the vaccine structure against the fab set at 0.05 IS, 0.15 IS and 0.3 IS. (**C)** The vaccine structure's charge heat map illustrates the accurate distribution of energy. (**D)** Charges of the vaccine structure against the fab set at 0.05 IS, 0.15 IS and 0.3 IS.

The secondary structural properties of κ‐helix, β‐strand and random coils were investigated using two separate servers, as shown in Table 4. In contrast, the servers predicted the frequencies of α‐helix, β‐strand and random coils to be 32.24%, 8. 41% and 47.78%, respectively, as seen in Figure 2. Also, CHARGE HEATMAP and ENERGY HEATMAP were shown in Figure 3. The sequence of the vaccine structure and its secondary structure were shown in Table S1 and Figure S1 of the supplementary material.

**TABLE 4:** The secondary structural properties of κ‐helix, β‐strand and random coils and MolProbity results of vaccine.

Row	Measurement index	Amino Acid	Percentage (%)
1	**Alpha helix (Hh)**	276	32.24%
2	**3_10_ helix (Gg)**	0	0.00%
3	**Pi helix (Ii)**	0	0.00%
4	**Beta bridge (Bb)**	0	0.00%
5	**Extended strand (Ee)**	99	11.57%
6	**Beta turn (Tt)**	72	8.41%
7	**Bend region (Ss)**	0	0.00%
8	**Random coil (Cc)**	409	47.78%
9	**Ambiguous states**	0	0.00%
10	**Other states**	0	0.00%
**MolProbity results of vaccine**			
1	**Row**	**Vaccine construct**	
2		**Percent**	**Details**
3	**MolProbity score**	2.17	—
4	**Clash score**	4.58	—
5	**Ramachandran favoured**	88.41%	—
6	**Ramachandran outliers**	2.17%	A318 THR, A325 ILE, A344 PRO
7	**Rotamer outliers**	2.86%	A240 SER, A362 ASP, A374 TYR
8	**C‐beta deviations**	4	A325 ILE, A297 ILE, A365 TYR, A343 THR
9	**Bad bonds**	0/1016	—
10	**Bad angles**	11/1377	A365 TYR, A344 PRO, A363 MET, A261 ASP, A324 HIS, A292 PHE, A374 TYR, A343 THR
11	**Cis non‐proline**	11/1377	(A367 ASP‐A368 ASN)
12	**Twisted non‐proline**	1/129	(A306 LEU‐A307 THR)
13	**Twisted prolines**	1/10	(A343 THR‐A344 PRO)

### 3.5 Verification and Validation of the Tertiary Structure

**FIGURE 4:**
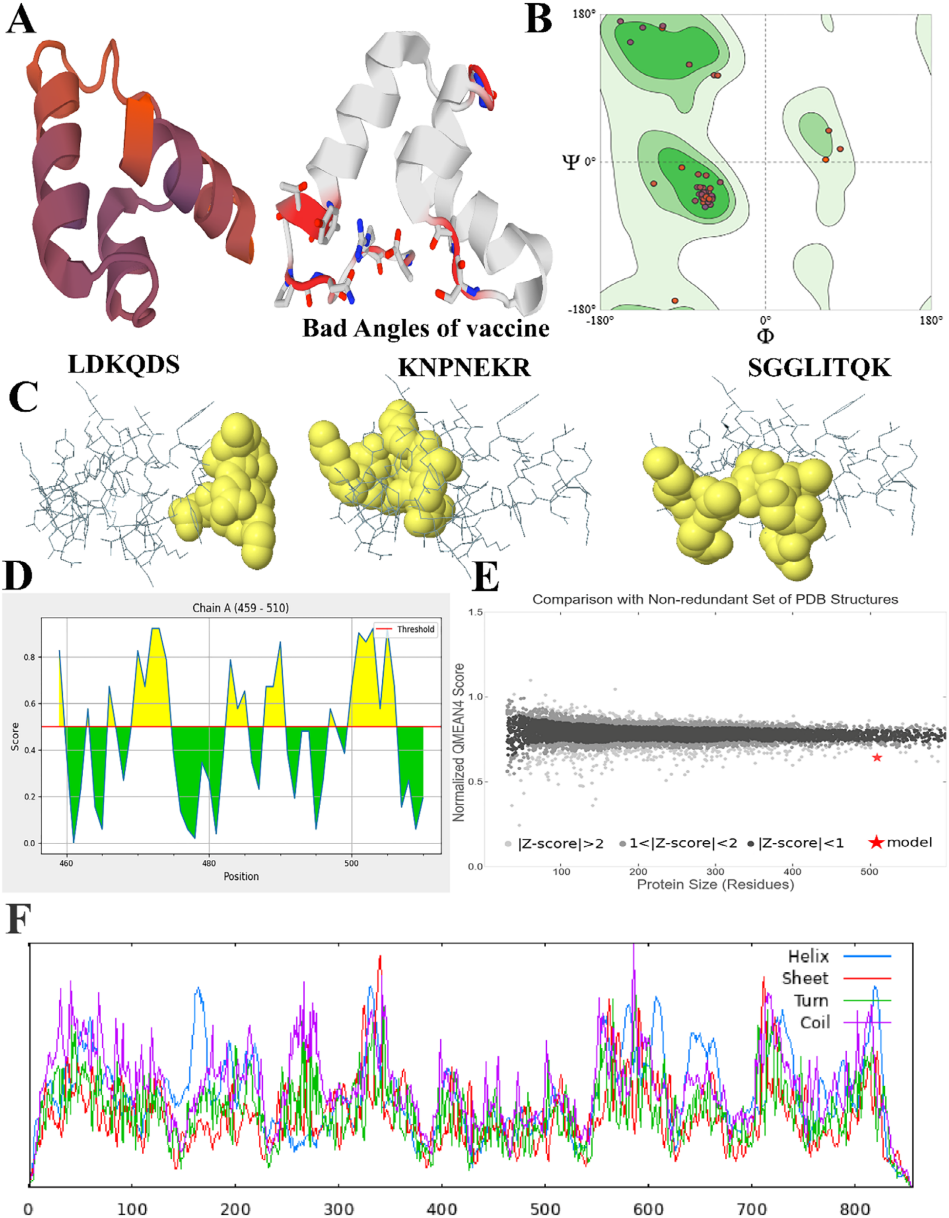
(A) The whole three‐dimensional configuration of the vaccination construct. (B) A Ramachandran plot explicitly developed for the vaccine. According to the Ramachandran plot, it was observed that 88.41% of the residues were situated inside the favoured zone. (C) Discontinuous epitopes on the vaccine's intended surface. (D) Discontinuous B cell epitopes were expected to have a particular significance. (E) The predicted structure was validated with a Z score <1. (F) Vaccine secondary structure prediction has been significantly improved using consensus prediction based on multiple alignments.

RaptorX was the optimal template for constructing the top five homology models. Among the five models, we opted for the one exhibiting the lowest C‐score, recorded as ‐4.97. Figure 4A displays the three‐dimensional depiction of the vaccine. Prior to refining, the Ramachandran plot of the vaccine indicated that 88.41% of residues were located in the most advantageous zone and 2.17% in the extra permissible area (Table 4). Figure 4B depicts the Ramachandran diagram illustrating the structure of the vaccine. The Z‐score of the refined model was ‐3.66.

### 3.6 Conformational B Cell Epitope

Three isolated B cell epitopes, with scores ranging from 0.601 to 0.753, were determined to consist of 21 residues. The size range for the conformation epitopes exhibited a variation between 459 and 510 residues. A score of 0.601 or higher was selected for the discontinuous peptides predicted by Ellipro, as shown in Figure 4C and Table S2. Figure 4D displays each interrupted epitope's score in the vaccination sequence, whereas Figure 4E displays the locations corresponding to the QMEAN local values in the B‐factor column. Figure 4F shows the alpha helix, beta sheets and random coils diagram well in the vaccine structure.

### 3.7 Disulfide Engineering in Vaccines

Disulfide engineering was used to stabilise the vaccine design. The DbD2 webserver discovered that 30 pairs of amino acids in our vaccination may form disulfide bonds. After accounting for other factors like energy and the chi 3 8score, it was suggested to make five pairs of mutations. The residue pairings with the most alterations were thus TYR461‐ TYR495, ALA464‐ LEU486, ALA465‐ ALA509, TYR468‐ PRO476 and ILE487‐ GLN491. The allowed values for energy, B‐factor and chi3 are shown in Table 5A. Also, Table 5B shows the number of the top 5 vaccine models according to the GalaxyWEB site.

**TABLE 5:** (A) The allowed values for energy, B‐factor and chi3 for prediction of Disulfide bonds of vaccine. (B) Structural information of the top 5 vaccine models.

(A)								
Res1 Chain	Res1 Seq #	Res1 AA	Res2 Chain	Res2 Seq #	Res2 AA	Chi3	Energy	Sum B‐Factors
A	461	TYR	A	495	TYR	102.18	0.64	1.25
A	464	ALA	A	486	LEU	−89.86	3.54	1.33
A	465	ALA	A	509	ALA	68.41	4.67	1.36
A	468	TYR	A	476	PRO	91.79	3.93	1.27
A	487	ILE	A	491	GLN	81.93	3.97	1.31

(B)						
Model	GDT‐HA	RMSD	MolProbity	Clash score	Poor rotamers	Rama favoured
Initial	1.0000	0.000	2.849	16.1	4.5	86.0
MODEL 1	0.9760	0.370	1.666	14.5	0.0	100.0
MODEL 2	0.9760	0.368	1.755	18.1	0.0	100.0
MODEL 3	0.9712	0.370	1.698	15.7	0.0	100.0
MODEL 4	0.9567	0.407	1.755	18.1	0.0	98.0
MODEL 5	0.9663	0.389	1.806	20.5	0.0	98.0

### 3.8 Molecular Docking

The vaccination (ligand) and TLR5 (receptor) were docked to forecast their binding affinity and interactions. Consequently, the ClusPro v2.0 web server generated eight docked complexes at different locations. Among the available options, we selected the complex with the lowest energy value and the binding position that showed functional interactions. Consequently, model 1 satisfied the demand criteria. Consequently, it was selected as the optimal vaccine‐TLR5 complex, with a negative energy score of ‐995.4.

**FIGURE 5:**
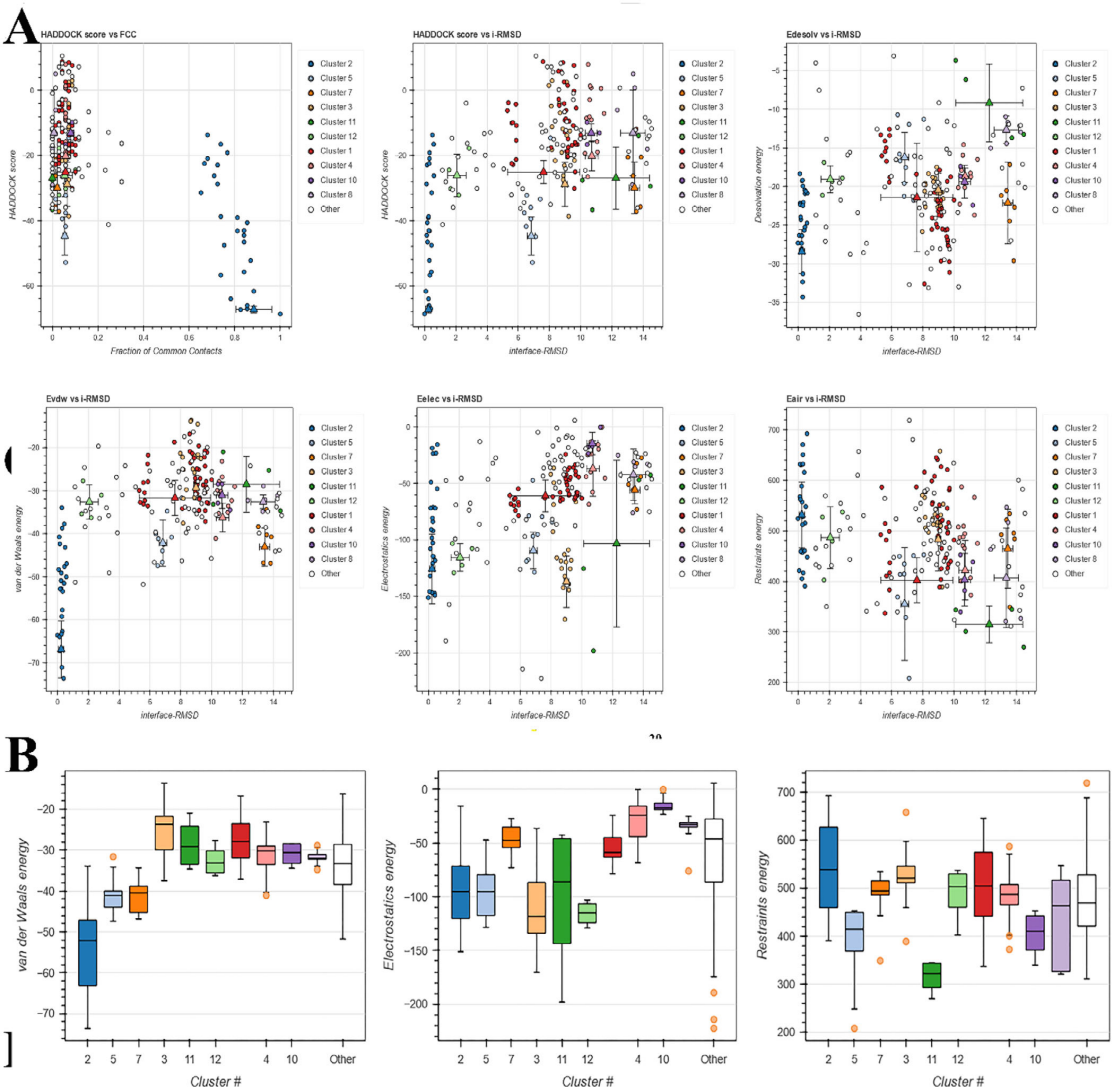
The statistics of the top 10 clusters of TLR5/Vaccine interaction. (A) Diagrams related to electrostatic energy, HADDOCK score, de‐solvation energy and restraints violation energy individually. (B) Graphs related to average electrostatic energy, Van der Waals energy and restraints violation energy. According to the results, Cluster 2 had the best molecular docking result between the vaccine structure and the TLR5 receptor.

The HADDOCK webserver's PRODIGY function was employed to investigate the docked complexes generated by ClusPro 2.0 and determine the binding affinity value (kcal/mol). In addition to ranking scores, HawkDock calculates the binding‐free energy (kcal/mol). The binding‐free energy was computed using the MM‐GBSA score on the HawkDock website server. HADDOCK clustered 144 structures in 12 cluster(s), which represents 72% of the water‐refined models HADDOCK generated. The statistics of the top 10 clusters are shown in Figure 5 and Table 6. The top cluster is the most reliable according to HADDOCK. Its Z‐score indicates how many standard deviations from the average this cluster is located in terms of score (the more negative the better). In the docking experiment performed using the ClusPro 2.0 and PRODIGY websites, the vaccine exhibited the highest binding affinity (39.23 kcal/mol) when docked with TLR‐5 (Figure 5A). Moreover, the vaccine exhibited the most significant outcomes when tested against the HawkDock server‐nominated TLR‐5 and the MM‐GBSA study, demonstrating a relative binding‐free energy of 46.24 (kcal/mol). An investigation was conducted on the binding connections and residues involved in the active site residues of the selected complex (Figure 5B).

**TABLE 6:** Cluster scores of docked vaccine TLR5 and vaccine complex.

Property	Cluster 2	Cluster 5	Cluster 7	Cluster 3	Cluster 11	Cluster 12	Cluster 1	Cluster 4	Cluster 10	Cluster 8
HADDOCK score	−67.3 ± 0.9	−44.8 ± 5.1	−29.9 ± 6.9	−28.8 ± 5.9	−26.9 ± 8.2	−26.1 ± 5.6	−25.1 ± 3.1	−20.1 ± 4.0	−13.1 ± 2.4	−13.1 ± 11.4
Cluster size	26	9	6	20	4	4	42	13	4	5
RMSD from the overall lowest‐energy structure	0.7 ± 0.5	3.8 ± 0.1	5.4 ± 0.1	5.3 ± 0.1	4.9 ± 0.6	3.0 ± 0.4	6.8 ± 0.4	7.3 ± 0.1	6.1 ± 0.3	5.3 ± 0.2
Van der Waals energy	−66.9 ± 5.8	−42.1 ± 4.7	−43.1 ± 3.8	−29.3 ± 2.5	−28.5 ± 5.6	−32.6 ± 3.4	−31.7 ± 3.5	−36.2 ± 2.9	−31.1 ± 2.7	−32.6 ± 1.4
Electrostatic energy	−125.7 ± 26.8	−109.5 ± 14.1	−55.8 ± 10.7	−137.0 ± 19.8	−103.3 ± 64.0	−115.6 ± 10.6	−61.0 ± 12.3	−36.9 ± 21.2	−14.6 ± 8.7	−42.3 ± 19.9
Desolvation energy	−28.4 ± 2.5	−16.2 ± 2.8	−22.1 ± 4.6	−20.4 ± 1.9	−9.2 ± 4.4	−19.1 ± 1.5	−21.4 ± 6.1	−18.7 ± 0.9	−19.4 ± 1.9	−12.7 ± 1.5
Restraints violation energy	531.3 ± 56.4	355.0 ± 96.9	464.5 ± 67.8	483.0 ± 54.4	314.6 ± 31.3	486.5 ± 53.2	402.0 ± 38.6	421.9 ± 51.1	403.1 ± 45.3	406.7 ± 85.2
Buried Surface Area	2468.4 ± 144.1	1718.7 ± 84.5	1340.7 ± 24.1	1269.3 ± 19.4	1282.0 ± 143.1	1375.6 ± 153.3	1273.3 ± 61.5	1227.7 ± 55.9	1178.9 ± 116.4	1157.3 ± 70.4
Z‐Score	−2.5	−1.0	0.0	0.0	0.2	0.2	0.3	0.6	1.1	1.1

### 3.9 Molecular Dynamic (MD) Simulation

**FIGURE 6:**
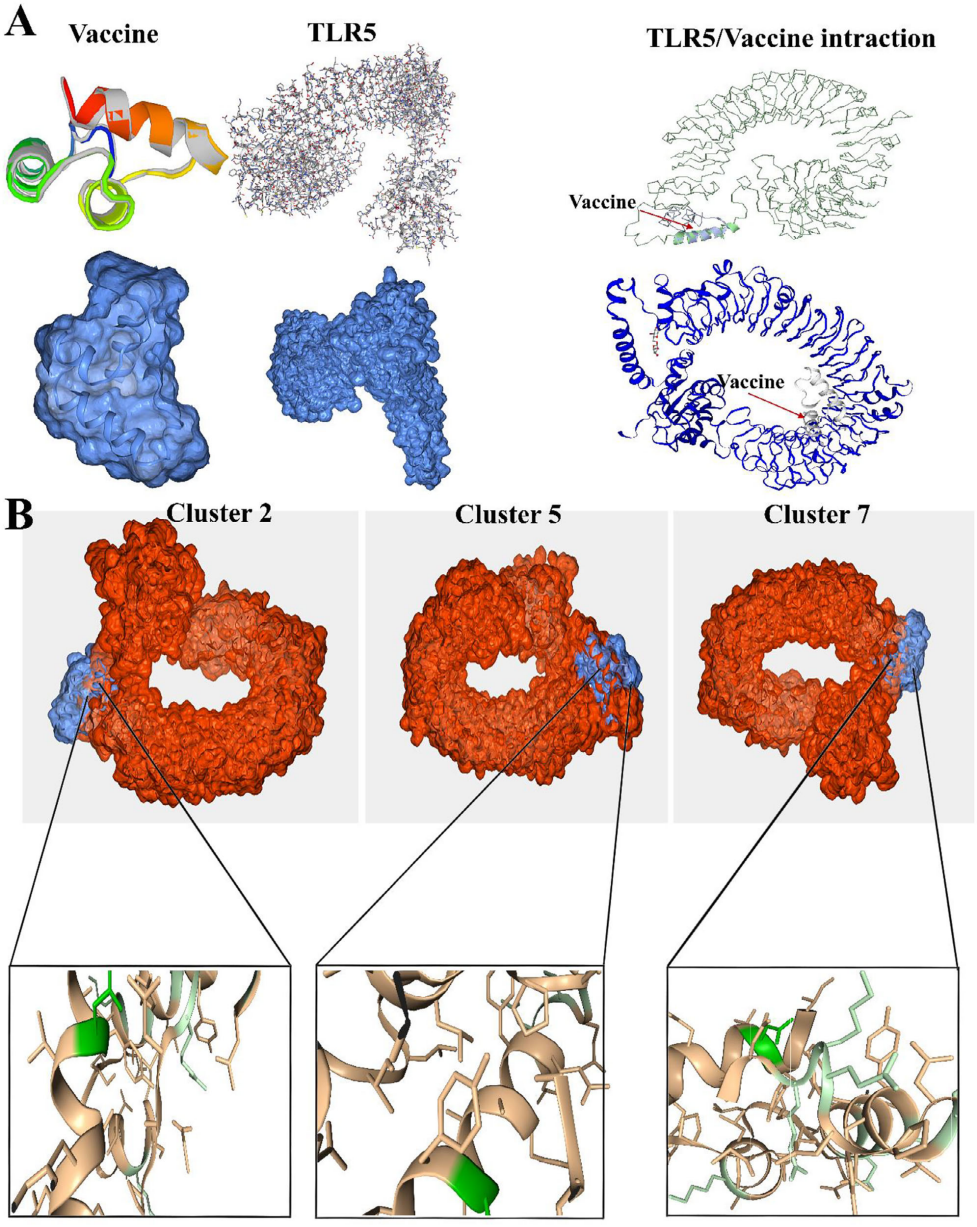
(A) The vaccine structure, TLR5 receptor and its alignment with the TLR5 receptor are all characterised by their three‐dimensional structure. (B) Molecular docking of vaccine structure and TLR5 receptor. The top 3 clusters were selected as the final constructs. All interacting residues from vaccine are shown in green colour.

The iModS software was used to simulate molecular dynamics. IModS analyses the structure by manipulating a complex force field at various intervals. The heat map exhibits a substantial co‐related region and a minimal root mean square deviation (RMSD), indicating robust interconnections among respective residues. Table 6 thoroughly explains the data obtained from the MD analysis (Figure 6).

### 3.10 Codon Adaptation and in Silico Cloning

**FIGURE 7:**
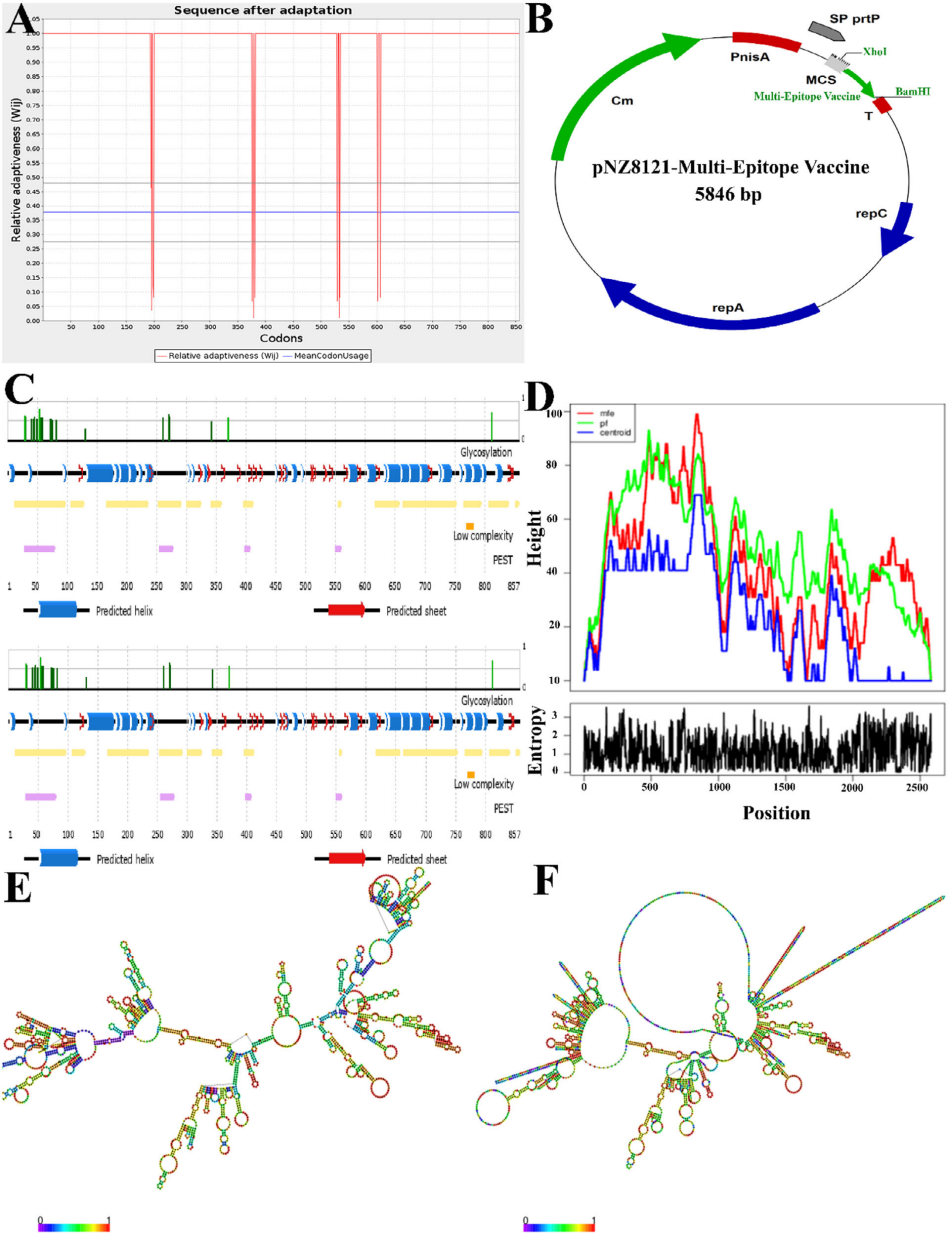
(A) The process of optimising codons in vaccine constructions targeting *Lactococcus lactis*. (B) Vaccine cloning using computational methods. The Green region inside the pNZ8123 expression vector represents the multi‐epitope vaccination insert. (C) The corresponding vaccine design map. (D) The mRNA entropy of the vaccine structure indicates the mRNA stability. (E, F) Different mRNA structures predicted from the vaccine structure.

In order to enhance the efficiency of vaccine translation, we modified the codons based on the *Lactococcus lactis* strain using the JCat service. The peptide used in the vaccine construct generated nucleotide sequences with a total length of 846 amino acids. In addition, the nucleotide sequence that has been altered exhibits a GC content of 37.34% and a CAI score of 0.95, correspondingly (Figure 7A). The modified sequence was inserted into the pNZ8124 vector using the XhoI and BamHI restriction locations as the initial and final cut points (Figure 7B). The corresponding vaccine design map was shown in Figure 7C. The improved vaccine construct has been inserted into the pNZ8124 cloning plasmid utilising the SnapGene program. The secondary structure of mRNA was predicted using the RNA fold server. The minimum free energy of ‐294.05 kcal/mol serves as an indicator of the thermodynamic stability of the mRNA structure. Furthermore, the first 12 nucleotides of the mRNA secondary structure exhibited a lack of pseudoknots or elongated stable hairpins, facilitating the optimal start of translation from the mRNA framework (Figure 7D). The MFE plain structure drawing and centroid plain structure drawing were used to predict two mRNA structures (Figure 7E). A mountain plot depiction of the MFE structure, the thermodynamic ensemble of RNA structures and the centroid structure are shown in this context. Furthermore, the positional entropy is shown for each location (Figure 7F).

## 4 Discussion

The global aquaculture industry is now susceptible to infectious illnesses triggered by *Lactococcus garvieae* and *Streptococcus iniae* (Hussein et al. 2023). This necessitates the use of an immunoinformatic approach to develop an epitope‐based vaccination. The vaccination using the pathogenic proteins showed exceptional relevance, as anticipated by immunoinformatics in prior research (Islam et al. 2022; Piri‐Gharaghie et al. 2022), confirming our endeavour's reliability. A vaccination provides safe and efficient protection against infectious diseases (Doerr and Berger 2014). It should be feasible to develop acquired immunity towards infectious illnesses (Netea et al. 2016). The management and prevention of infection and transmission caused by *Lactococcus garvieae* and *Streptococcus iniae* pose significant challenges without a viable vaccine (Halimi et al. 2020).

Moreover, efficient vaccination strategies are pending to address the present circumstances. The investigation yielded the development of a vaccine using epitopes intended to elicit a robust immune response against *Lactococcus garvieae* and *Streptococcus iniae* (Tsai et al. 2013). A new vaccine development approach is urgently required to address the economically hazardous aquaculture issue caused by *Lactococcus garvieae* and *Streptococcus iniae* (Imtiaz et al. 2023).

Our objective was to create an epitope vaccine targeting the virulent extracellular protein of *Lactococcus garvieae* and *Streptococcus iniae* since these proteins are crucial in immunological colonisation and transmission (Nguyen et al. 2018). All the pathogenic proteins chosen by in silico screening were assessed for their antigenic area to facilitate the recognition of this protein by humoral immune responses. The first stage was the identification of all potential LBL epitopes (Islam et al. 2022). Subsequently, vaccines were developed using antigenic LBL epitopes since the linkers below aligned with the foremost epitopes. LBL epitopes were employed in creating our peptide vaccine as a crucial element that enhances its stability, bending, and transcriptional regulation (Nezafat et al. 2014). Attachment of adjuvants to LBL epitopes using EAAAK sequences as linkers increases the vaccine's stability and durability while augmenting humoral immunogenic reactions (Piri‐Gharaghie et al. 2023).

The vaccine construction included amino acid residues. Solubility, a physicochemical feature, is a crucial attribute of a recombinant vaccine (Khatoon et al. 2017). A solubility assessment technique was employed to ascertain the vaccine construct's solubility inside the host's body, and the findings indicated its solubility. The produced vaccine had a fundamental character, as the theoretical PI level shows. According to the guidance provided by web server instruments, the stability index of the vaccination sequence suggests that it is expected to exhibit stability after the synthesis process.

In contrast, the vaccine's GRAVY score and aliphatic index suggested its hydrophobic nature and thermostability. Based on the projected physicochemical features and comprehensive parameter scores, it is very likely that this vaccine exhibits suitability as a potential option for combating *Lactococcus garvieae* and *Streptococcus iniae*. After evaluating the discovered models, the model with the lowest energy value was selected as the best, followed by the 3D structure projection based on the c‐score. In the validation procedure of the 3D structure, we observed the Z‐score and superior aspects of the most preferred, acceptable and forbidden regions for the Ramachandran plot. The molecular docking analysis indicated that the vaccine‐TLR5 complexes had the lowest energy score, suggesting potential infection‐inhibiting activity and intense interaction with these receptors (Islam et al. 2022). TLR5 in Teleosts exhibits sensitivity to flagellin protein. Consequently, flagellin peptides were used in the formulation of epitope‐based vaccines. Our findings indicate that these peptides can elicit an inflammatory reaction and foster the development of innate immune systems in fish (Gao et al. 2018; Su and Chen 2021). The application of molecular dynamics simulation has promise as a valuable tool for comprehending the functioning of proteins and the derivation of their structure (Salo‐Ahen et al. 2020).

Protein dynamic simulations can temporally induce anatomical movement. Dynamic simulations of the vaccine candidate were conducted for 50 nanoseconds, and the outcomes were assessed utilising the RMSD and RMSF values. The RMSD score is used to compare different atomic conformations of a molecular system. The RMSD score was used to evaluate vaccination candidates' significant flexibility and departure from receptor structure. On the other hand, the RMSF of the complex structure was used to determine the displacement of our specific vaccine candidate's atoms from the receptor structure.

Finally, an immunological simulation investigated the ideal target clearance and cell density parameters, resulting in the most effective immunologic reaction versus the pathogen (Rapin et al. 2010). The immune system generates memory B cells, which have a half‐life of many months due to the increased vaccination doses (Akkaya et al. 2020). The vaccine effectively replicated a humoral immune response by enhancing the generation of immunoglobulins. A molecular dynamics (MD) simulation was conducted to assess the stability of the multi‐epitope vaccination candidate with the receptor. Codon optimisation was performed to ensure the durability of the vaccine construct inside the host. In due course, the codon underwent modifications, leading to the successful in silico cloning of the desired vaccine candidate into the pNZ8124 vector, which serves as the expression host for *Lactococcus lactis*.

## 5 Conclusion

Despite extensive research into the structures of *Lactococcus garvieae* and *Streptococcus iniae*, controlling the diseases they cause has remained challenging due to their high mutation rates. To address this, our study employed a range of computational methodologies to pinpoint potential B cell epitopes within the virulent antigens of both bacteria. We then integrated these identified epitopes into a novel epitope‐based vaccine.

The designed vaccine demonstrated the desired immunodominant characteristics, showing a strong ability to bind to immunological receptors and elicit a significant immune response against bacterial infection. Our research suggests that selecting a suitable vaccine candidate, like the one we've developed, is a crucial first step in creating an effective vaccine to combat *Lactococcus garvieae* and *Streptococcus iniae* outbreaks in fish. The potential epitopes we've identified are also promising for further investigation.

However, to fully validate our findings, wet laboratory experiments are essential to definitively demonstrate the efficacy of our developed vaccine against *Lactococcus garvieae* and *Streptococcus iniae*. This will be the next critical step in translating our computational insights into a practical solution.

## Author Contributions

**Ramesh Ranjbar**: methodology, validation, writing—review and editing, writing—original draft, Investigation. **Abbas Doosti**: investigation, methodology, validation, writing—review and editing, writing—original draft, formal analysis, supervision. **Mostafa Shakhsi‐Niaei**: conceptualisation, software, formal analysis, resources, writing—review and editing, writing—original draft.

## Ethics Statement

The study was approved by the Ethics Committee of the Islamic Azad University of Shahrekord Branch in Iran (IR.IAU.SHK.REC.1400).

## Peer Review

The peer review history for this article is available at https://www.webofscience.com/api/gateway/wos/peer‐review/10.1002/vms3.70465.

## Supporting information




**Supporting Table 1**: The sequence of the vaccine structure.
**Supporting Table 2**: Predicted Discontinuous Epitope(s) of vaccine.
**Supporting Fig 1**: secondary structure of vaccine.

## Data Availability

The datasets analyzed during the current study are available from the corresponding author upon reasonable request.
