# Supplementary material for: Antigenic Protein Screening and Design of Multi‐Epitope Vaccine Against Lactococcus garvieri and Streptococcus iniae for Combating Lactococcosis and Streptococcosis in Fish
Source: Vet Med Sci. 2025 Jun 17;11(4):e70465. doi: 10.1002/vms3.70465 (PMC12172566; doi:10.1002/vms3.70465)
Supplement: Supplementary file 1 — Supporting Table 1: The sequence of the vaccine structure. Supporting Table 2: Predicted Discontinuous Epitope(s) of vaccine. Supporting Fig 1: secondary structure of vaccine. [file VMS3-11-e70465-s001.docx]

**supplementary material.**

## Table S1. The sequence of the vaccine structure

| Epitope composition | Vaccine sequence | | AlgPred | | SOLpro (<0.4) | VaxiJen |
| --- | --- | --- | --- | --- | --- | --- |
| Multi epitope | MGFFYEAAAKLDSETEIQSSSSATSDKDNTSSPQESELETAPTDTGQTTEAENSTDQTSEEVAPIEEEEPTTSETSEQEKTVEDKPKSEKITPADLSSDASYTDLPGAHKPDRKKTVKDKPVAAKKVSTTKSTAAY**AYLSEREKQEY**YELVEEIEGWQALEEEYAADLEEERAQSESSSPERRSRSSRSLDAAPAAFLSQRGKKKESRRAEENTNDETSEETASKKPKKKKRWIKAAYASTVFAADGGVYNSKKPASGPTDPVDPGDPGTTVDPEGPKNPGTDGKKASNFNFGTQEITSADKKKTLTDKTTRKKNRGTLAGKKHIVSASDAAADQSVASVTLTPGTSSKKAGKGAAAYGSMDMTYTDNANATGYEGYPLFSSSCLNQEHFYLEPLHEGQEPEASADKSKVYENSDKKIDDEDTGAKLYYTDPHNQKRYDTKKVKPDTVKKEKAGVDPQITYDTAAYLYQLDKQDSPSLGKLVSGGLITQKQADSYNDYYTKNPNEKRNAAYGSMDMTYTDNANIAGYEGYPLFSSSCLNQDHFYLEPLRQTSEPEANADKAKVYTNNKKLTIDYMANSAILKSMANDPQNYLEVPADNVGAGKTYLSDLLLEAAAKQKTRVKMHKSGKRKKSESISESTSESLSESISESAAYTSTLKKETVSAVSEKAAYKTDSDQARWKKNKENKKKTDQKGKAKYKKKVEDLPKGKKQIAVPNDATNEKKVTENKKDLKKASQTARSLTKKDKTSNGAAYIGQDPHEYEPLPEDVEKTTKKKKTKNKKKDQEAKSAFDKIPDAAYGQAGADPTYKKANETSTATNQEAVTAAYQNEKGHGKKQTDQYGIFGYKKEVTAPTSEV | NON ALLERGEN | | 0.643 | | **0.8547** |
| KK (LINKER FOR EACH PROTEIN EPITOPS)  AAY (LINKER BETWEEN DIFFERENT PROTEINS)  EAAAK (LINKER BETWEEN DIFFERENT BACTERIA and adjuvant)  GFFY (adjuvant) | | | | | | |

**Table S2. Predicted Discontinuous Epitope(s) of vaccine**

| No. | Residues | Number of residues | Score |
| --- | --- | --- | --- |
| 1 | A:L470, A:D471, A:K472, A:Q473, A:D474, A:S475 | 8 | 0.753 |
| 2 | A:K501, A:N502, A:P503, A:N504, A:E505, A:K506, A:R507 | 7 | 0.717 |
| 3 | A:S483, A:G484, A:G485, A:L486, A:I487, A:T488, A:Q489, A:K490 | 6 | 0.601 |


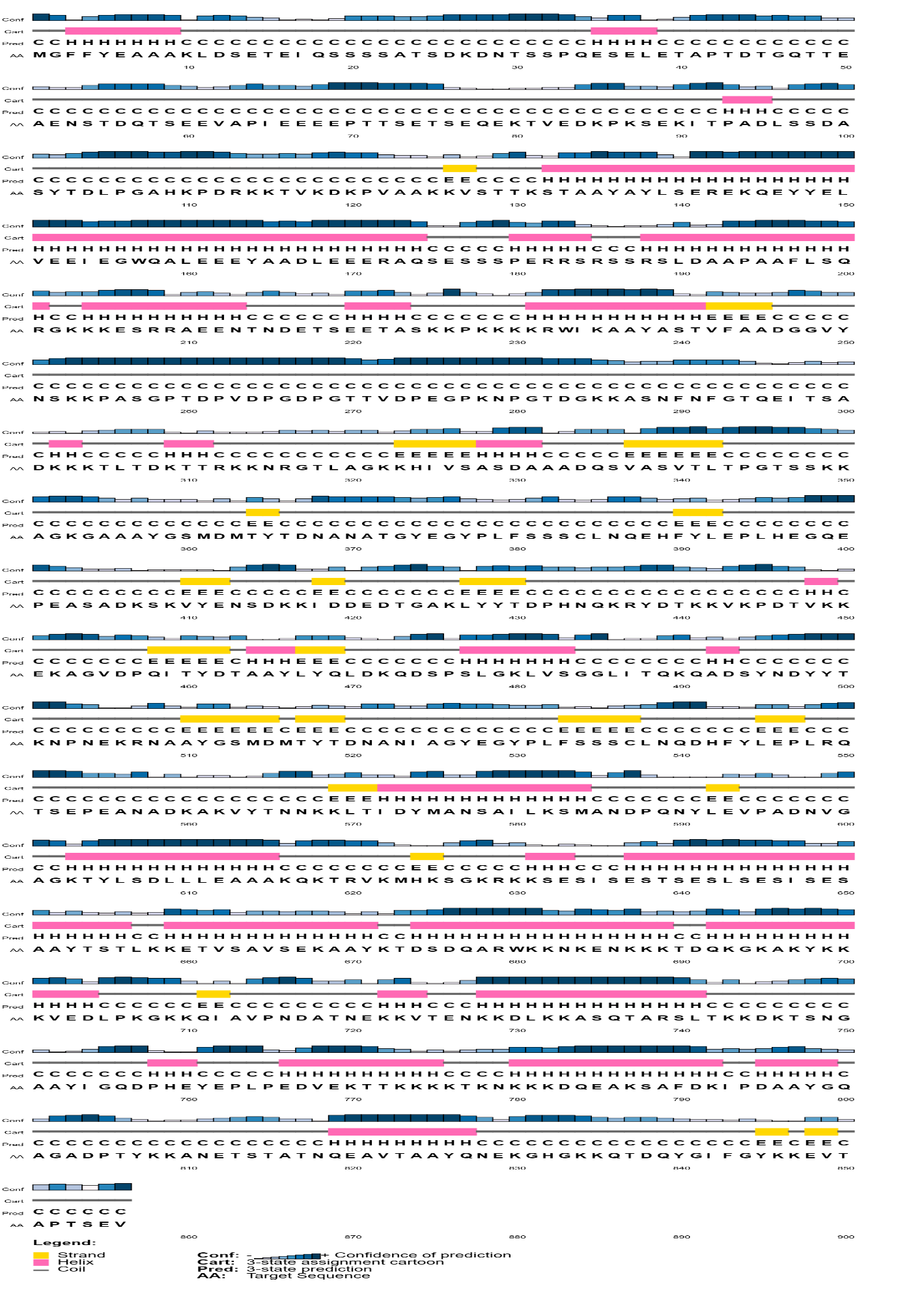


**Figure S1.** **secondary structure of vaccine**
